# Convenient Preparation and Spectroscopic Characterization of 7*R*-Hydroxymatairesinol

**DOI:** 10.3390/molecules26195838

**Published:** 2021-09-26

**Authors:** Eleonora Colombo, Giuseppe Paladino, Umberto Ciriello, Daniele Passarella

**Affiliations:** 1 Dipartimento di Chimica, Università degli Studi di Milano, 20133 Milano, Italy; eleonora.colombo@unimi.it; 2 Linnea S.A., 6595 Riazzino, Switzerland; gpaladino@linnea.ch (G.P.); uciriello@linnea.ch (U.C.)

**Keywords:** 7-hydroxymatairesinol (HMR), *allo*-hydroxymatairesinol, lignans, *Picea abies*

## Abstract

The preparation of 7*R*-HMR (*allo*-hydroxymatairesinol) is reported by: (a) NaBH_4_ kinetic reduction of 7*R*/7*S* diastereomeric mixture; and (b) epimerization of the C7 hydroxyl group by Mitsunobu reaction and subsequent ester hydrolysis. The availability of highly pure target compound (7*R*-HMR) made it possible to confirm the structure of the target compound and to complete the full spectroscopic characterization.

## 1. Introduction

Lignans are a large group of low molecular weight polyphenols, derived by oxidative coupling of monolignols. They are plant secondary products and present many different biological activities including anticancer, antioxidant, antimicrobial, anti-inflammatory, and immunosuppressive [1]. Many plant lignans are constituents of human nutrition, present in flax and sesame seeds in high concentrations and in whole grain cereals, beans, other vegetables, some fruits and berries, wines, tea, and coffee in lower concentrations [2]. Until recently, flaxseeds were considered one of the richest sources of lignans. However, Norway spruce (*Picea abies*) branch stubs (or knots) have been shown to be extremely rich in them [3]. They contain on average about 10% by weight of lignans of which (−)-7*S*-hydroxymatairesinol (7*S*-HMR) makes up 70–85% [4]. 7*S*-HMR occurs not only in spruce knots but is the dominant lignan in cereals—such as in wheat, triticale, oat, barley, millet and corn bran—and in amaranth whole grain [5]. Its presence in cereals was not detected in previous studies because of inadequate analytical methods using either acidic or alkaline treatment steps where 7*S*-HMR is converted primarily to conidendrin or conidendric acids, respectively [3]. Methods for the separation of spruce knots and isolation of 7*S*-HMR have been developed, making it the most readily available lignan [6].

7*S*-HMR has been shown to metabolize to (−)-enterolactone by intestinal bacteria in rats and humans and to have chemopreventive effects on the development of DMBA induced mammary carcinoma in rats [7,8]. It has also been shown to have a chemopreventive effect in the ApcMin mice model of human familial adenomatous polyposis [9]. Furthermore, it has been shown to be a strong antioxidant and able to reduce the oxidation of LDL-particles in vitro [8]. It has been marketed as a nutritional supplement since 2006 [10].

7*S*-HMR is accompanied by a stereoisomer differing in stereo-chemistry at C7. The major isomer is (7*S*, 8*R*, 8′*R*)-(–)-7-hydroxymatairesinol while the minor is (7*R*, 8*R*, 8′*R*)-(–)-hydroxymatairesinol (7*R*-HMR), that is reported also as *allo*-HMR. In the average extract the 7*S*/7*R* ratio is around 3:1. In the literature, no full characterization and full assignment of the minor stereoisomer is reported due to its difficult separation from its main diastereomer. We were thus interested in an efficient synthesis of 7*R*-HMR to obtain it in a pure form and complete its characterization.

In this paper, two approaches to the obtainment of pure 7*R*-HMR are presented: the first is an enrichment of 7*R*-HMR taking advantage of the different kinetic of reduction of the two diastereomers, while the second is an epimerization of the hydroxylic group in position C7, de facto converting 7*S*-HMR into 7*R*-HMR by Mitsunobu reaction. The full characterization and assignment of 7*R*-HMR is reported.

## 2. Results

### 2.1. Enrichment of 7R-HMR (***2***) in the Extract Mixture

**Scheme 1:**
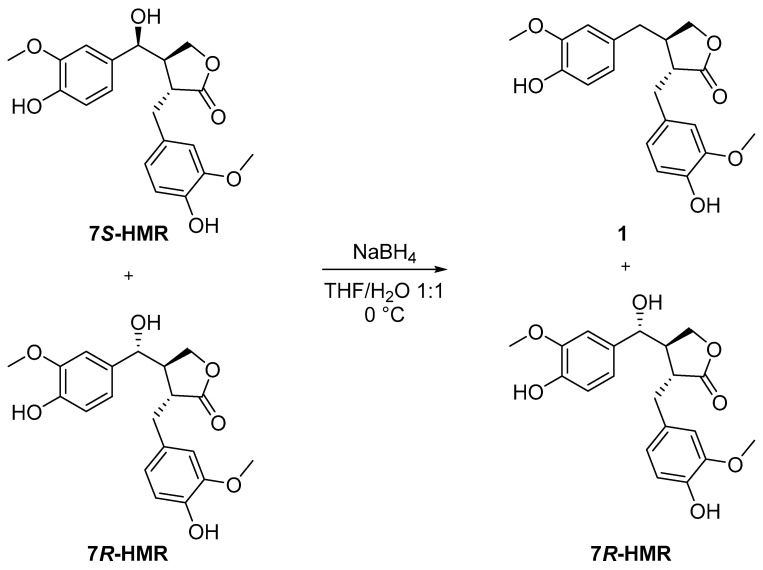
Enrichment of *7R*-HMR by hydride reduction.

The used starting mixture contained a ratio of 7*S*-HMR/7*R*-HMR around 2:3.8. This derives by repeated extraction/purification processes of 7*S*-HMR of the plant extract. As a difference in the kinetic of reduction between the two diastereomers is known [3], the reaction of said mixture with NaBH_4_ was attempted (Scheme 1). 7*S*-HMR was expected to be almost quantitatively transformed to (−)-matairesinol (**1**), whereas the other isomer (7*R*) should show only partial conversion at the same conditions. Several attempts were carried out in order to only obtain 7*R*-HMR and 7*S*-HMR in the reaction mixture, that can be more easily separated.

**Figure 1:**
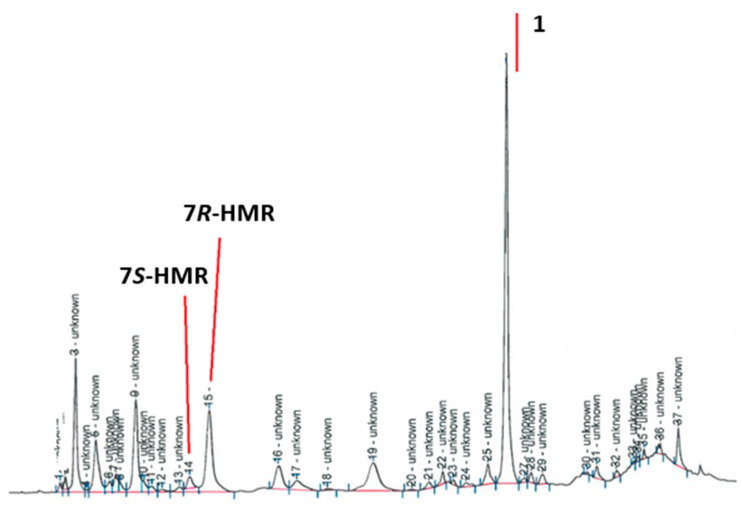
HPLC chromatogram relative to the reaction after 3.5 h.

The best attempt using around 0.7 g of substrate led after 4 h to a mixture of 7*S*-HMR (1.2%), 7*R*-HMR (12.5%), and **1** (31.1%). The mixture was purified through successive chromatographies, until the obtainment of 100 mg of a 7*R*-HMR that contains around 6% of 7*S*-HMR. The reaction was monitored by HPLC (Table 1 and Figure 1) that gave us the possibility to perfectly distinguish the peaks of the target compounds. The optimized conditions were then applied for a reaction on larger scale of substrate (54 g) and the results appeared consistent with the small-scale reaction. The main problem of this procedure is the appearance of unknown byproducts that compromise the purification process and requires several repeated purification steps.

### 2.2. Conversion of 7S-HMR in 7R-HMR

**Scheme 2:**
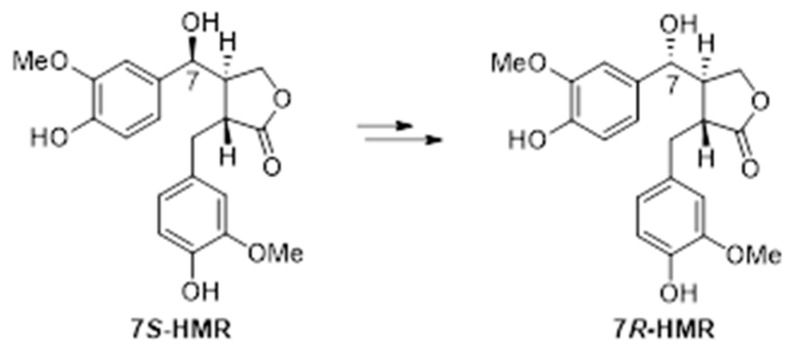
Conversion of 7*S*-HMR into 7*R*-HMR by Mitsunobu strategy.

In the second approach we developed an inversion of the C7 configuration using modified Mitsunobu conditions (Scheme 2).

**Scheme 3:**
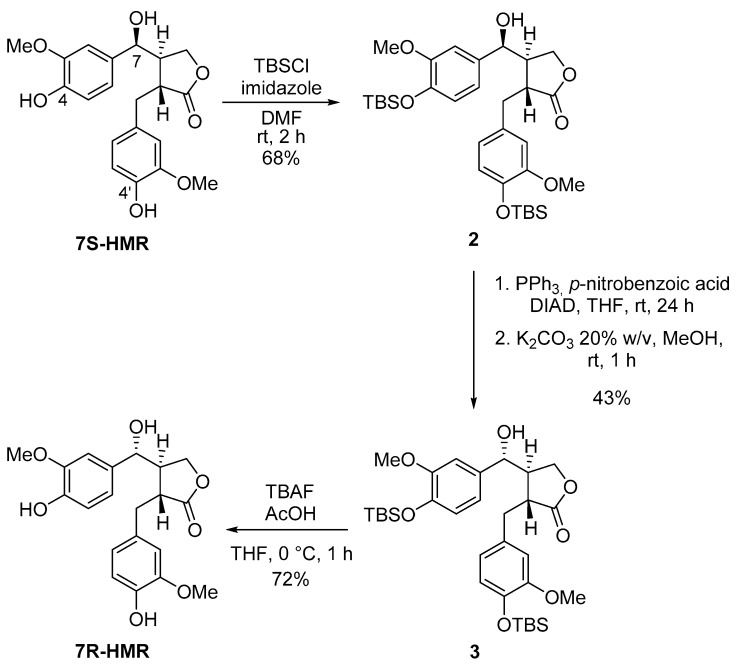
Synthetic strategy for preparation of 7*R*-HMR.

Below (Scheme 3) reports the procedure for the preparation of desired compound 7*R*-HMR, performed optimizing the strategy previously reported by Fischer et al. [11].

Starting from 7*S*-HMR the first reaction we performed was the selective protection of the phenolic hydroxyl groups at C4 and C4′ to block their acidic behavior that could interfere with the subsequent Mitsunobu reaction. The reaction took place at room temperature and the exact control of the temperature was found to be crucial to avoid the contamination of compounds deriving from not selective undesired reactions. The second step involves the inversion of the C7 hydroxy group of 7*S*-HMR exploiting the efficacy of Mitsunobu reaction. This reaction was carried out with diisopropyl azodicarboxylate (DIAD) that is considered safer than diethyl azodicarboxylate (DEAD). The obtained ester of the *p*-nitrobenzoic acid was then hydrolyzed to guarantee the obtainment of the corresponding secondary alcohol with the desired configuration at position C7. The formation of the ester intermediate was evaluated by ^1^H-NMR analysis, appearing to be a single compound. In particular, a downfield shift of proton H-7 signal (δ 5.8 ppm, doublet) was observed, proving the inversion of configuration at C7. In the final step for the preparation of compound 7*R*-HMR, we removed the TBS protecting groups. Once again, the control of the reaction conditions and in particular the control of the temperature resulted crucial for the success of the reaction. The use of the conditions reported in the literature [11] gave very low yield with insuperable problems in the purification process. Decreasing the temperature and in particular reaching 0 °C, we obtained compound 7*R*-HMR in good yields (72%) and the purification process by flash chromatography gave compound 7*R*-HMR with appreciable purity. We also had the opportunity to confirm that the initial purity of the target compound is compromised by the in solution epimerization due to the thermodynamic stability of the 7*S* diastereoisomer.

### 2.3. Spectroscopic Characterization

Spectroscopic data for the obtained (−)-7*R*-HMR are reported below (Table 2).

**Figure 2:**
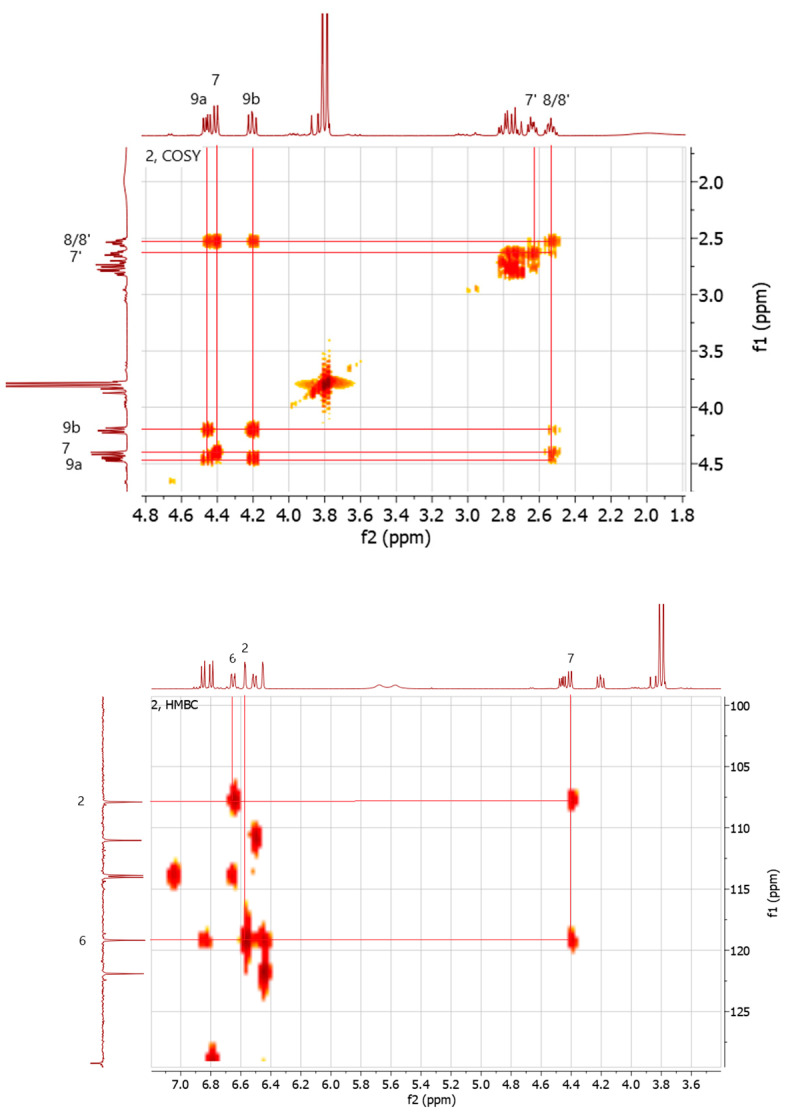
Most relevant COSY and HMBC interactions for 7*R*-HMR structure elucidation.

The obtained compound has been purified by chromatography and then submitted to spectroscopic characterization in order to confirm the target structure. All the ^1^H- and ^13^C-NMR signals were assigned on the base of COSY, HSQC, and HMBC spectra. The crucial signal is the one at 4.40 ppm (d, *J* = 7.8 Hz) that is due to H-7 and its corresponding ^13^C C7 signal at 74.4 ppm (HETCORR spectrum). By HMBC spectrum, it is possible to identify the signal of carbons C2 (107.9 ppm) and C6 (119.3 ppm) with the corresponding protons (6.57 and 6.65 respectively). With the use of COSY spectrum the signals of H-5, the corresponding C5 (by HETCORR) and the spin system of the other phenyl group were identified and assigned. H-7 ^1^H signal appears connected (COSY) with the signal at 2.48–2.57 ppm (H-8). The crosspeaks of H-8 confirmed the presence of the signals due to H_2_-9 at (4.46 and 4.20 ppm) and the signal due to H-8′ (2.49–2.58 ppm). H-8′ appears directly connected (COSY) with H_2_-7′ (2,68–2,83 ppm). Some of these interactions are shown below (Figure 2). The HETCORR made the assignment of all the corresponding carbon signals possible. See Supplementary Materials for the complete spectra.

The NMR spectra are strictly related to the ones of the corresponding C7 epimer (7*S*-HMR) used as starting material. In particular, the chemical shift of the signal due to H-7 moves from 4.63 (7*S*-HMR) to 4.40 ppm (7*R*-HMR) and this evidence confirms the different interactions of H-7 within the space of the molecule deriving from the inversion of the configuration and the obtainment of the diastereoisomer 7*R*. The evaluation of the purity of the obtained compounds is monitored by HPLC analysis (Discovery HSF5 25 cm × 21.2 mm × 10 μm) with an eluent composed by two solutions 78:22 respectively composed by H_2_O with 0.1% HCOOH and CH_3_CN/MeOH (3:7) 0.1% HCOOH and using 20 mL/min flux. The same conditions have been used for semipreparative purification of limited quantity of 7*S*-HMR.

## 3. Materials and Methods

Plant extracts, 7*S*-HMR/7*R*-HMR mixture and pure 7*S*-HMR have been furnished by Linnea SA (Via Cantonale CH, 6595 Riazzino (TI), Switzerland).

^1^H-NMR spectra were recorded on Bruker DRX-400 And Bruker DRX-300 instruments and are reported relative to residual CDCl_3_. ^13^C-NMR spectra were recorded on the same instruments (101 and 75 MHz) and are reported relative to CDCl_3_. Chemical shifts (δ) for proton and carbon resonances are quoted in parts per million (ppm) relative to tetramethylsilane (TMS), which was used as an internal standard.

HR-ESI mass spectra were recorded on FT-ICR APEXII (Bruker Daltonics, Billerica, MA, USA). Specific rotations were measured with a P-1030-Jasco polarimeter with 10 cm optical path cells and 1 mL capacity (Na lamp, λ = 589 nm).

### 3.1. Reduction Reaction of a Diasteroisomeric Mixture of 7S- and 7R-HMR

0.766 g (2.04 mmol) of 7*S*-HMR/7*R*-HMR around 2:3.8 were dissolved in a 1:1 mixture of THF and H_2_O (40 mL). The mixture was cooled at 0 °C and NaBH_4_ (0.389 g, 10.2 mmol) was added portion wise, the reaction was then monitored by HPLC (see Table 1). After leaving it overnight at rt, the reaction was quenched by adding aqueous 10% HCl (3.5 mL) until reaching pH ≈ 4–5 and the mixture was extracted with CH_2_Cl_2_. The organic layer was washed with H_2_O, dried over Na_2_SO_4_, filtered, and evaporated under reduced pressure to obtain a brown oil (530 mg).

### 3.2. Reduction of 7S-HMR on Multigram Scale

50.45 g (134.9 mmol) of 7*S*-HMR/7*R*-HMR around 2:3.8 were dissolved in a 1:1 mixture of THF and H_2_O (500 mL). The mixture was cooled at 0 °C and NaBH_4_ (12.63 g, 333.9 mmol) was added portion wise, the reaction was then monitored by HPLC (Table 3). After leaving it for 4 h at 0 °C the reaction was quenched by adding aqueous 10% HCl until reaching pH ≈ 5 and the mixture was extracted with CH_2_Cl_2_ (2 × 250 mL). The organic layer was washed with H_2_O (2 × 100 mL), dried over Na_2_SO_4_, filtered and evaporated under reduced pressure to obtain a brown oil (29.52 g).

Silica chromatography purification: 10 g of the reaction mixture were submitted to purification through gravimetric silica chromatography (SiO_2_ 120 g). Dry loading (SiO_2_ ≈ 12 g), column diameter = 3.5 cm, h = 24 cm, 60 mL fractions. Eluent: gradient from CH_2_Cl_2_:MeOH 100:1 to 25:1. 7*R*-HMR was obtained (1.7 g) with 89% purity (11% is due to 7*S*-HMR).

Subsequent HPLC preparative columns are performed: Column: Discovery HSF5 25 cm × 21.2 mm × 10 μm. FMA:H_2_O + 0.1% HCOOH-FMB: ACN/MeOH (3:7) + 0.1% HCOOH-Premixed (2.0 L) FMA/FMB 78/22, flux: 20 mL/min. 850 mg of starting mixture gave 100 mg of 7*R*-HMR with 94.2% purity (5.8% is due to 7*S*-HMR)

### 3.3. Preparation of (−)-bis-TBS-7S-HMR (***2***)

TBSCl (0.420 g, 2.79 mmol) and imidazole (0.453 g, 6.65 mmol) are added to a solution of (−)-7*S*-HMR (0.500 g, 1.34 mmol) in DMF (5.1 mL) at rt.

The reaction mixture was stirred for 4 h then washed with saturated aqueous NaHCO_3_ and brine and dried over anhydrous sodium sulfate. The solvent was removed in vacuo and the crude material was purified by flash chromatography (Hexane/EtOAc 8:2) to give title compound as colorless oil (0.550 g, 68% yield).

^1^H-NMR (400 MHz, CDCl_3_): δ 6.81 (d, *J* = 8.1 Hz, 1H), 6.78–6.44 (m, 5H), 4.58 (d, *J* = 6.8 Hz, 1H), 3.92–3.83 (m, 2H), 3.78 (s, 3H), 3.75 (s, 3H), 3.13 (dd, *J* = 13.2, 4.9 Hz, 1H), 3.07–2.97 (m, 1H), 2.94–2.78 (m, 1H), 2.62–2.54 (m, 1H), 0.97 (s, 9H), 0.95 (s, 9H), 0.13 (s, 6H), 0.11 (s, 6H) ppm.

^13^C-NMR (100 MHz,CDCl_3_): δ 179.3, 151.3, 150.9, 145.3, 143.8, 135.0, 131.1, 122.1, 121.0 120.7, 118.4, 113.6, 109.9, 75.2, 68.2, 55.5, 55.4, 45.1, 43.8, 35.0, 25.7, 18.4, −4.6 ppm.

${\left[a\right]}_{D}^{20}$ = −1.58 (c 1, CHCl_3_).

HR-ESI-MS: MW 625.2993 calcd. for C_32_H_50_O_7_Si_2_Na, MW 625.3001 found.

### 3.4. Preparation of (+)-bis-TBS-7S-HMR (***3***)

DIAD (0.5 mL, 2.58 mmol), p-nitrobenzoic acid (0.431 g, 2.58 mmol) and triphenylphosphine (0.677 g, 2.58 mmol) were added to a solution of the TBS protected 7*S*-HMR (**2**) (0.519 g, 0.860 mmol) in anhydrous THF (21.5 mL) at rt. The mixture was stirred at rt overnight then the solvent was removed in vacuo. The p-nitrobenzoate was purified by flash chromatography (Hexane/AcOEt 8:2) then dissolved in methanol (7.5 mL) and treated with an aqueous solution of K_2_CO_3_ (0.36 mL, 20% *w/v*). After 1 h, the mixture was diluted with diethyl ether and washed with saturated aqueous NH_4_Cl. The organic phase was separated, and the aqueous phase was extracted with diethyl ether. The combined organic phases were dried over anhydrous sodium sulfate and the solvent was removed in vacuo. The product was purified by flash chromatography (Hexane/AcOEt 8:2) to give title compound as a colourless oil (0.225 g, 43%).

^1^H-NMR (300 MHz, CDCl_3_): δ 6.79 (d, *J* = 8.1 Hz, 1H), 6.72 (d, *J* = 7.8 Hz, 1H), 6.65 (d, *J =* 2.0 Hz, 1H), 6.60–6.58 (m, 2H), 6.47 (dd, *J =* 8.1, 2.0 Hz, 1H), 4.39 (dd, *J* = 6.6, 2.2 Hz, 1H), 4.29 (dd, *J =* 9.3, 7.1 Hz, 1H), 3.98 (dd, *J* = 8.1, 8.1Hz, 1H), 3.77 (s, 3H), 3.74 (s, 3H), 2.85–2.67 (m, 3H), 2.58–2.47 (m, 1H), 0.98 (s, 9H), 0.97 (s, 9H), 0.12 (s, 6H), 0.11 (s, 6H) ppm.

^13^C-NMR (100 MHz, CDCl_3_): δ 180.0, 151.3, 151.0, 145.1, 143.9, 135.1, 130.9, 121.5, 121.0, 120.7, 118.1, 112.9, 109.5, 74.1, 67.9, 55.4, 46.1, 43.3, 34.7, 25.7, 18.4, −4.6 ppm.

${\left[a\right]}_{D}^{20}$ = + 8.11 (c 0.50, CHCl_3_).

HR-ESI-MS: MW 625.2993 calcd. for C_32_H_50_O_7_Si_2_Na, MW 625.3004 found.

### 3.5. Preparation of (−)-7R-HMR

To a solution of the (+)-bis-TBS-7*R*-HMR (**3**) (1.12 g, 1.86 mmol) in THF (50 mL) and acetic acid (0.41 mL, 7.44 mmol) was added TBAF (7.44 mL, 1M in THF, 7.44 mmol) at 0 °C. The reaction mixture was stirred at 0 °C for 2 h, then washed with saturated aqueous NH_4_Cl and extracted with diethyl ether. The organic extracts were combined and the solvent was removed in vacuo. The residue was purified by flash chromatography (Hexane/AcOEt 1:1), giving 7*R*-HMR as a colorless oil (0.500 g, 72%).

^1^H-NMR (400 MHz, CDCl_3_): δ 6.82 (d, *J* = 8.0 Hz, 1H), 6.76 (d, *J =* 7.9 Hz, 1H), 6.63 (dd, *J =* 8.1, 1.9 Hz, 1H), 6.54 (d, *J* = 1.9 Hz,1H), 6.47 (dd, *J =* 8.0, 1.9 Hz, 1H), 6.42 (d*, J =* 1.9 Hz, 1H), 5.67 (bs, 1H), 5.57 (bs, 1H), 4.43 (dd, *J =* 9.5, 5.6 Hz, 1H), 4.38 (d, *J =* 7.8 Hz, 1H), 4.22 (dd, *J =* 9.5, 7.6 Hz,1H), 3.79 (s, 3H), 3.74 (s, 3H), 2.79–2.72 (m, 2H), 2.65–2.59 (m, 1H), 2.54–2.47 (m, 1H), 1.98 (bs, 1H) ppm.

^13^C-NMR (100 MHz, CDCl_3_): δ 179.0, 146.9, 146.7, 145.7, 144.8, 134.0, 129.3, 121.9, 119.3, 114.0, 113.9, 111.0, 107.9, 74.4, 68.4, 55.7, 46.2, 35.0 ppm.

${\left[a\right]}_{D}^{20}$ = − 3.45 (c 0.10, CHCl_3_).

7*S*-HMR ${\left[a\right]}_{D}^{20}$ = − 13.3 (c 4, THF)

HR-ESI-MS: MW 397.1263 calcd. for C_20_H_22_O_7_Na, MW 397.1271 found.

## 4. Conclusions

The use of two different approaches to obtain 7*R*-HMR gave us the opportunity to confirm—with high accuracy—the structure of the target compound and to have enough high purity compound available to complete its full and detailed spectroscopic characterization.

## Figures and Tables

**Table 1:** HPLC monitoring of the reduction reaction after 15 h.

	Area%		
	7*S*-HMR (rt = 19.7 min)	7*R*-HMR (rt = 21.7 min)	1 (rt = 51.3 min)
20 min	12.60	20.95	1.30
50 min	9.59	19.20	1.47
2 h	5.17	13.76	1.39
3 h	4.59	19.61	1.57
4 h	1.2	12.5	31.1
15 h	0	0	27.76

**Table 2:**
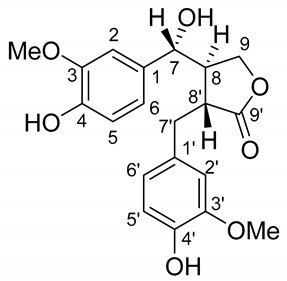
NMR signal assignment for 7*R*-HMR and comparison with 7*S*-HMR chemical shift.

	^7*R*-HMR^				^7*S*-HMR^	
	^1^H		*J* (Hz)	^13^C	^1^H	^13^C
*1*				134.0		133.4
*2*	6.57	d	1.8	107.9	6.67	108.2
*5*	6.85	d	8.1	114.0	6.85	114.4
*6*	6.65	dd	8.1 1.8	119.3	6.69	118.8
*7*	4.40	d	7.8	74.4	4.63	75.3
*8*	2.49–2.57	m		46.2	2.61	45.1
*9*	4.46	dd	9.5 5.5	68.4	3.90–4.02	68.6
	4.20	dd	9.4 7.4			
*1* *′*				129.3		129.5
*2* *′*	6.45	bs		111.0	6.61	111.9
*3-OCH_3_*	3.78	s		55.7	3.81	55.9
*3′-OCH_3_*	3.81	s			3.85	
*5* *′*	6.79	d	7.9	113.9	6.79	114.0
*6′*	6.50	dd	7.9 1.9	121.9	6.60	122.5
*7′*	2.68–2.83	m		35.0	2.90–3.05	35.1
*8′*	2.49–2.58	m		46.2	2.90–2.98	43.7
*9′*				179.0		179.3
*3*				146.9		146.8
*4*				145.7		146.6
*3′*				146.7		145.5
*4′*				144.8		144.4
*3-OH*	5.57	b			5.55	
*3*′*-OH*	5.67	b			5.65	
*7-OH*	1.98	b			1.91	

**Table 3:** HPLC monitoring of the reduction reaction after 3.5 h.

Time	Area%		
	7*S*-HMR	7*R*-HMR	1
2 h	4.59	19.61	n.d.
3.5 h	1.2	12.5	31.09

## Data Availability

Not applicable.

## Supplementary Materials

Spectroscopic data relative to 7*R*-HMR, compounds **2** and **3** are available online, Figure S1: ^1^H-NMR relative to 7*R*-HMR, Figure S2: COSY relative to 7*R*-HMR, Figure S3: HSQC relative to 7*R*-HMR, Figure S4: HMBC relative to 7*R*-HMR, Figure S5: ^13^C-NMR relative to 7*R*-HMR, Figure S6: ^1^H-NMR relative to compound **2,** Figure S7: ^13^C-NMR relative to compound **2,** Figure S8: ^1^H-NMR relative to compound **3,** Figure S9: ^13^C-NMR relative to compound **3**.

## Sample Availability

Samples of the compounds are available from the authors.
