# Supplementary material for: Convenient Preparation and Spectroscopic Characterization of 7R-Hydroxymatairesinol
Source: Molecules. 2021 Sep 26;26(19):5838. doi: 10.3390/molecules26195838 (PMC8512340; doi:10.3390/molecules26195838)
Supplement: Supplementary file 1 [file molecules-26-05838-s001.zip › molecules-1373120-supplementary.pdf]

Convenient Preparation and Spectroscopic Characterization of 7*R*-Hydroxymatairesinol

Eleonora Colombo[1], Giuseppe Paladino,[2] Umberto Ciriello[2] and Daniele Passarella[1,*]

Supplementary Materials

- ^{1}H, COSY, HSQC, HMBC and ^{13}C NMR spectra of compound **7*R*-HMR**;
- ^{1}H and ^{13}C NMR of compound **2**;
- ^{1}H and ^{13}C NMR of compound **3**;

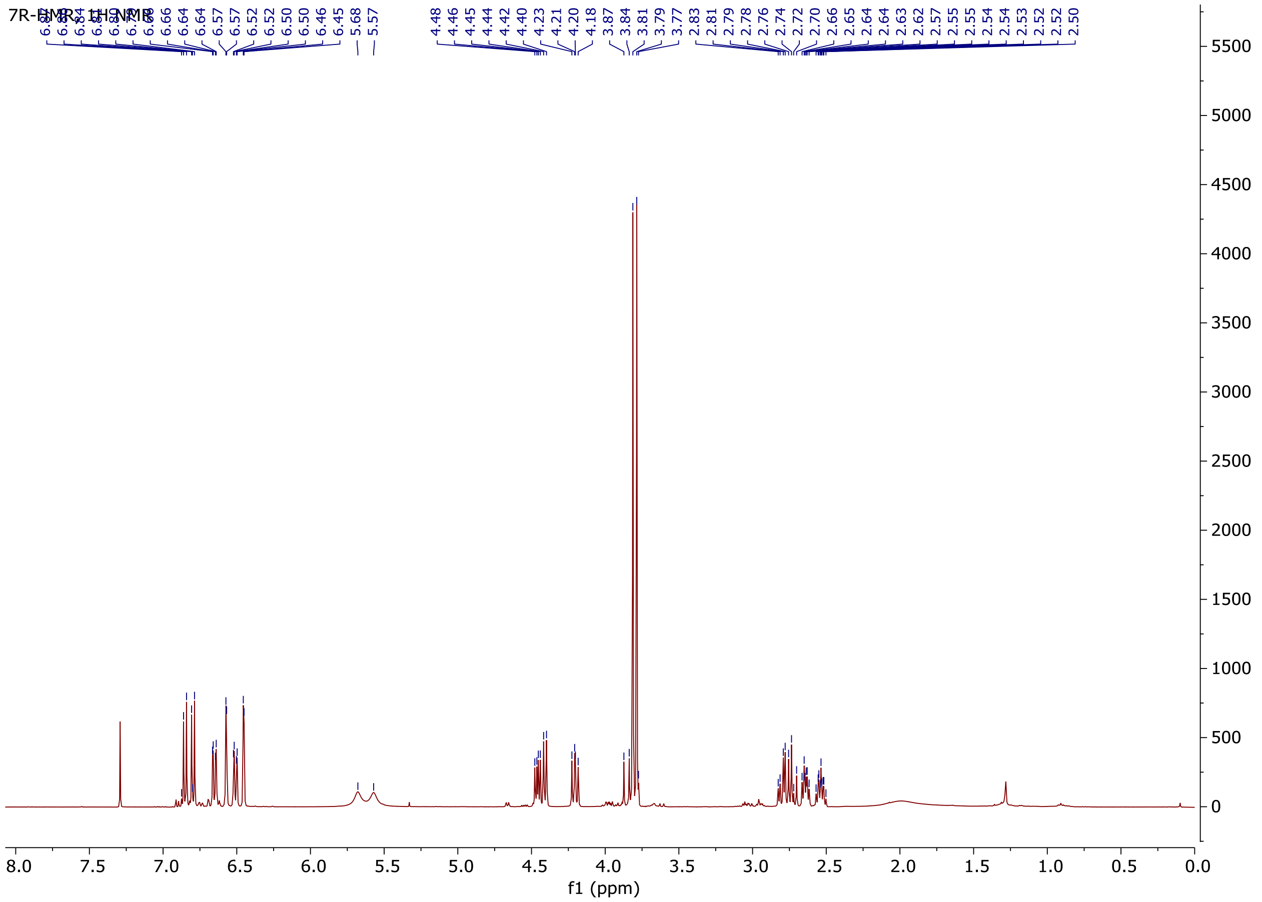

Figure S1. ^{1}H-NMR relative to 7R-HMR.

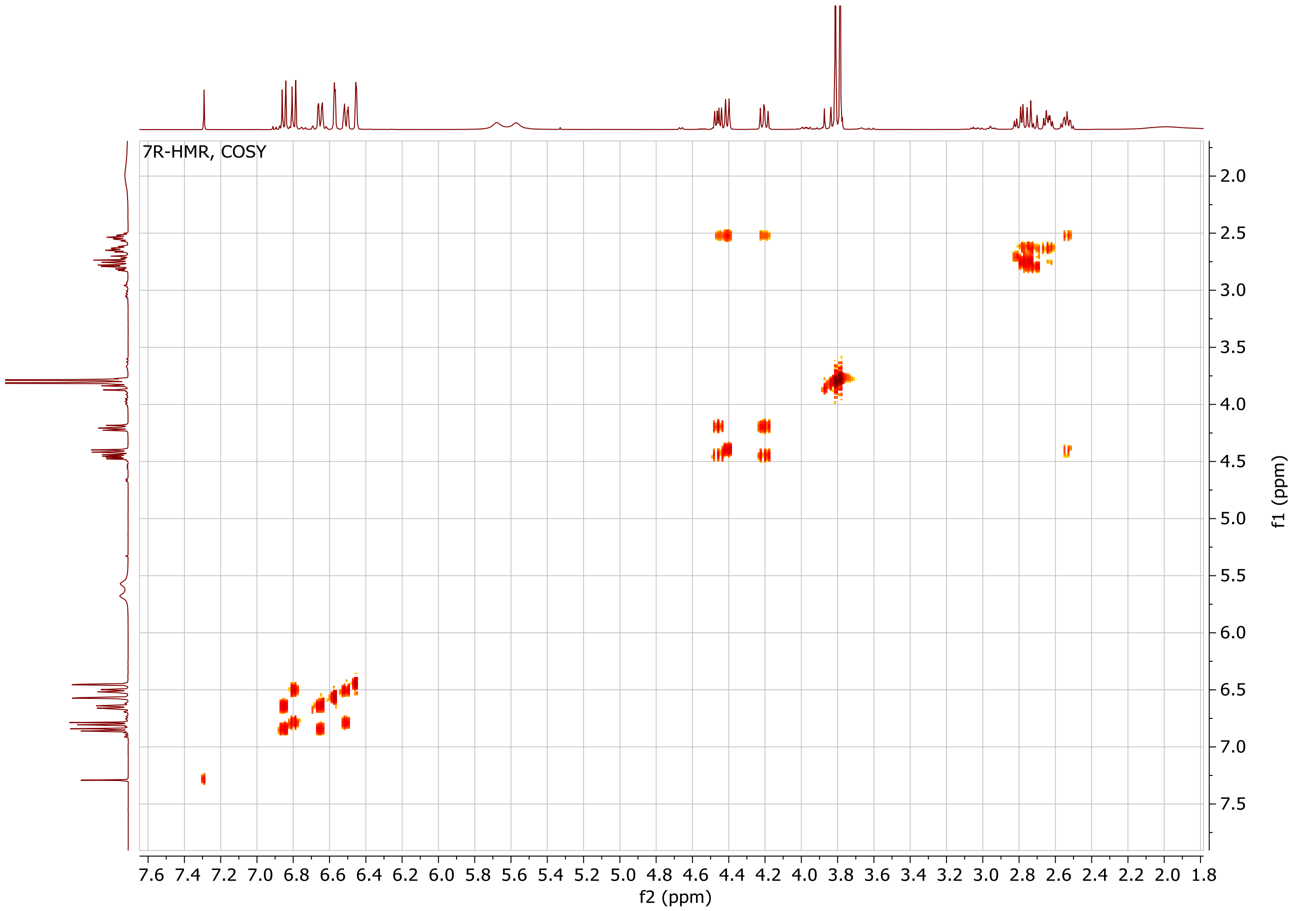

Figure S2: COSY relative to 7R-HMR.

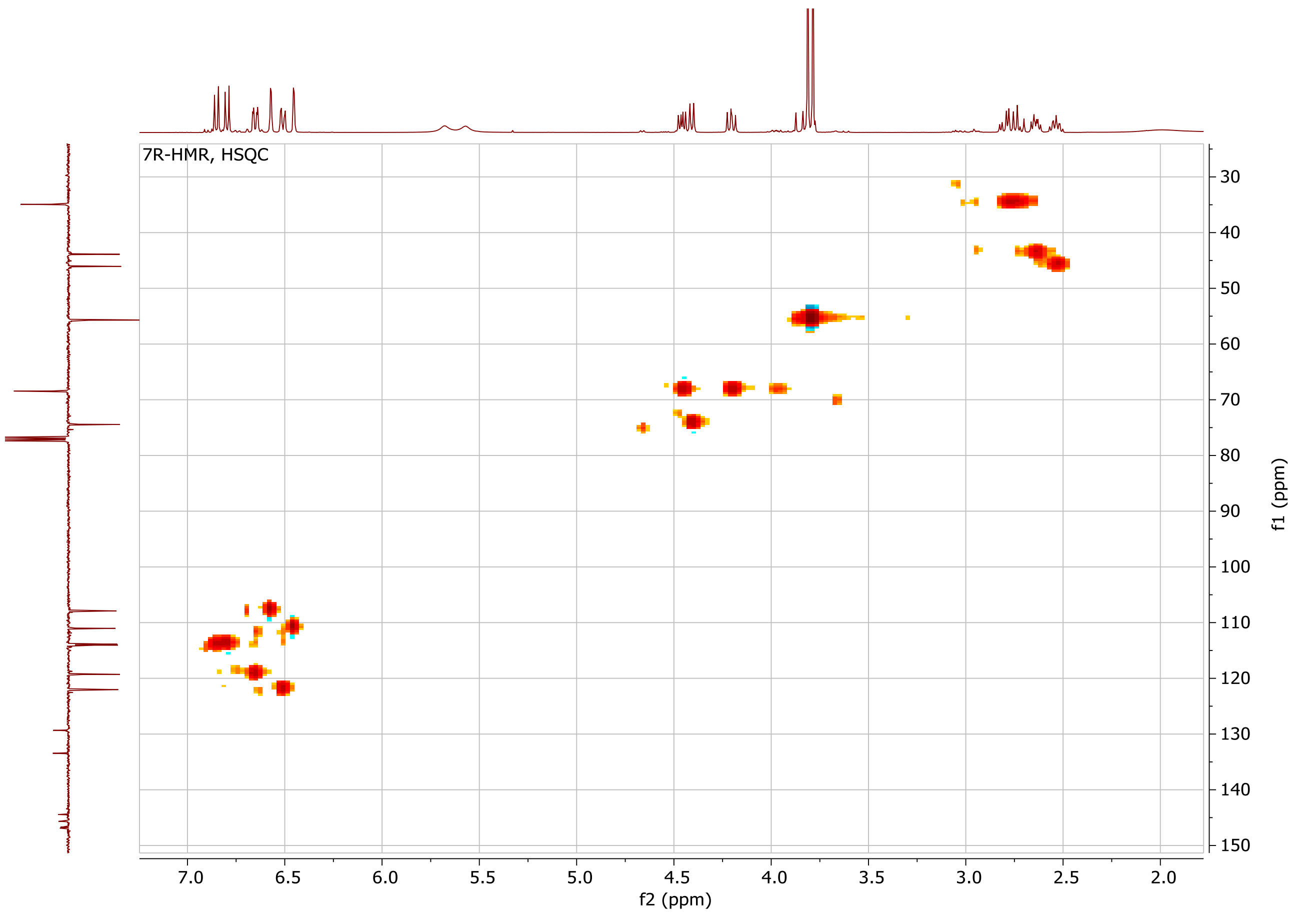

Figure S3: HSQC relative to 7R-HMR.

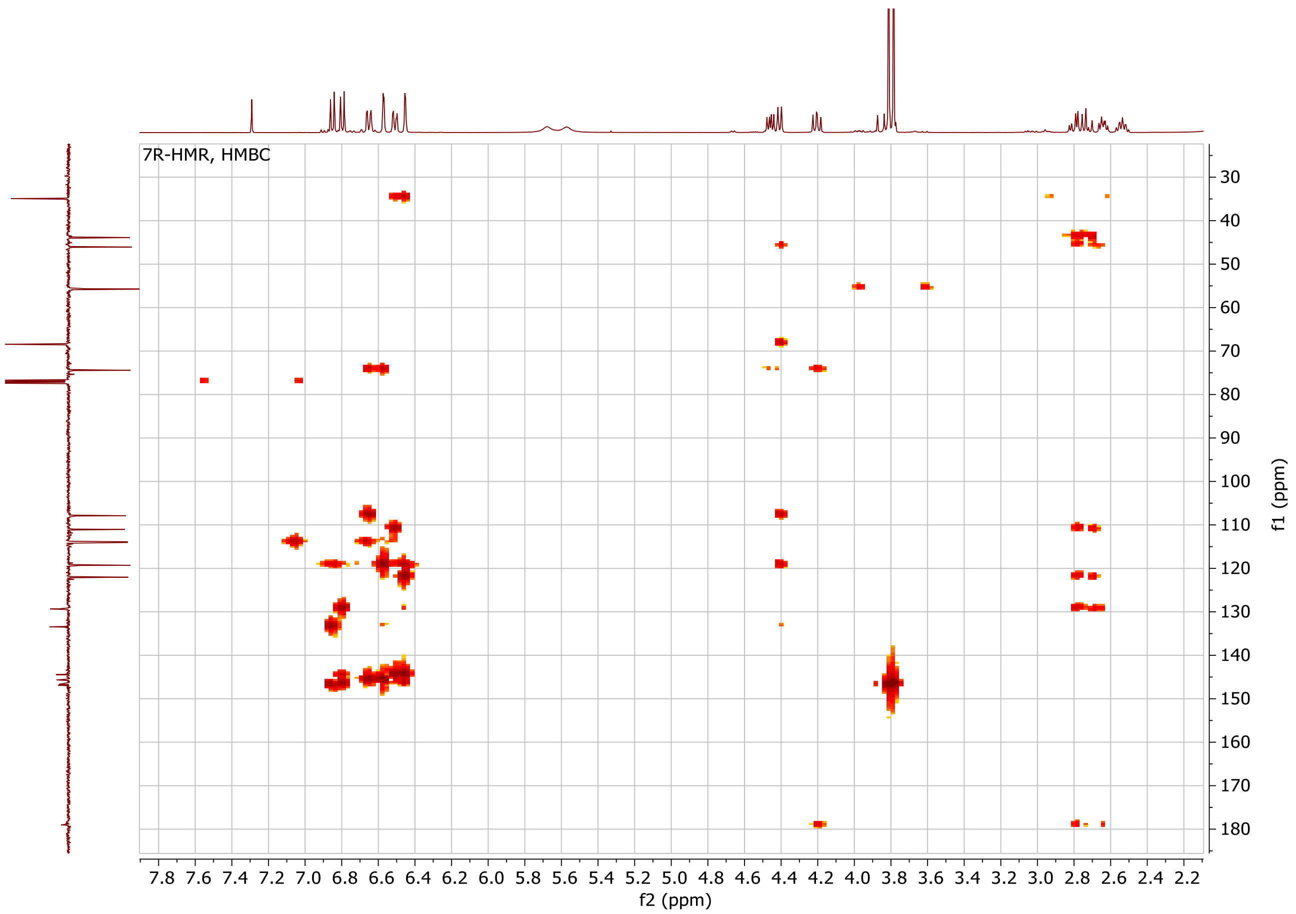

Figure S4: HMBC relative to 7R-HMR.

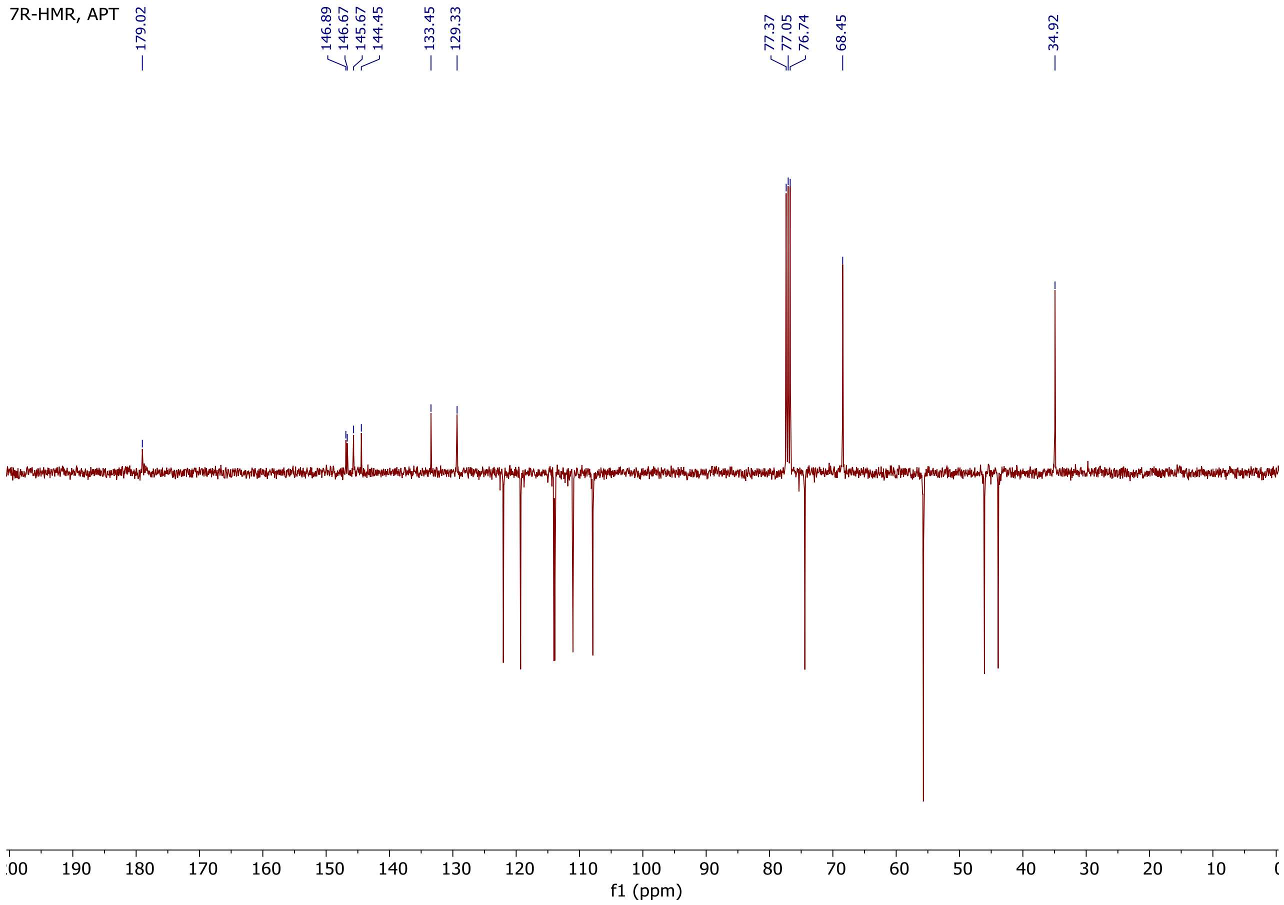

Figure S5. ^{13}C-NMR relative to 7R-HMR.

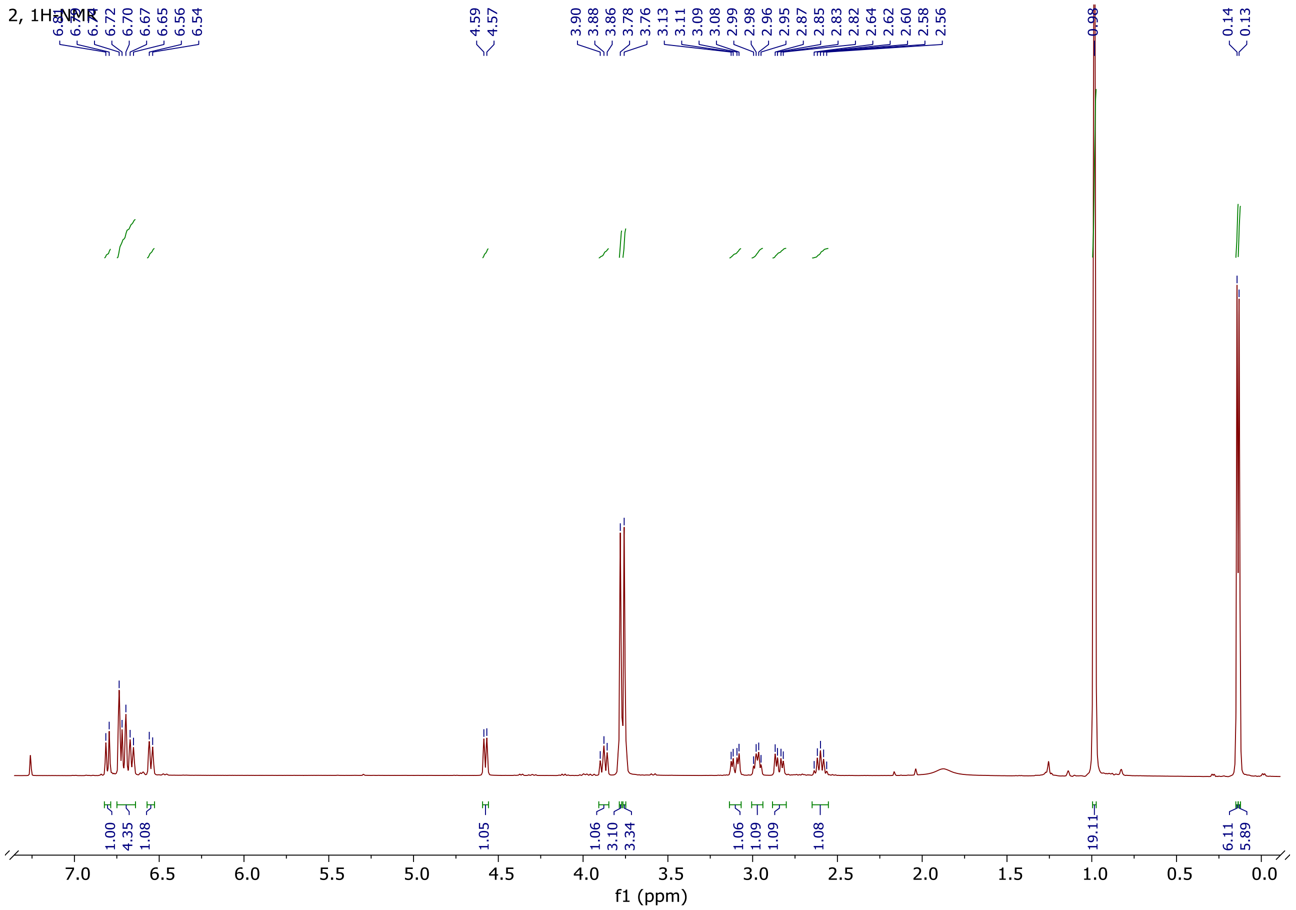

*Figure S6: ^{1}H-NMR relative to compound **2**.*

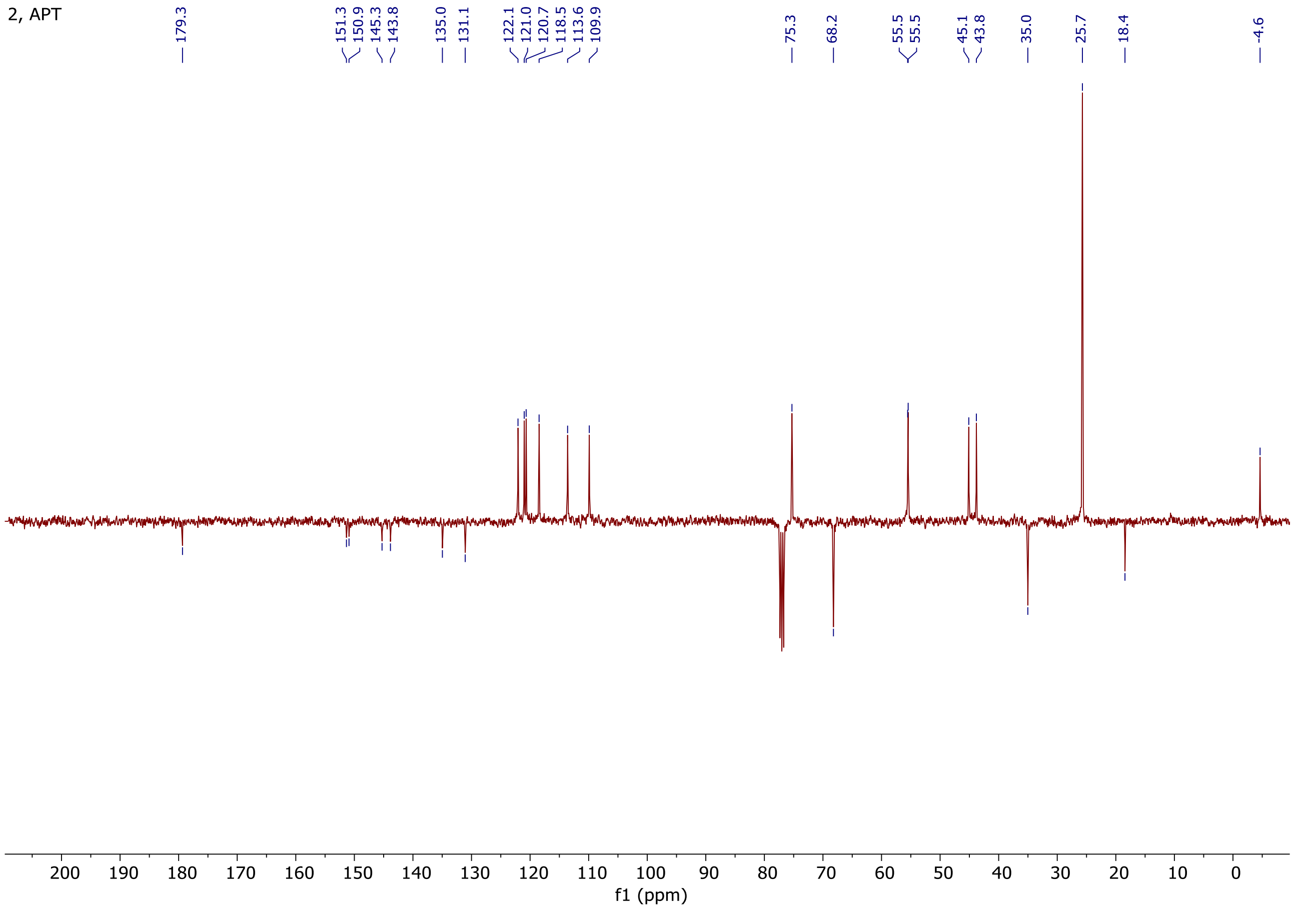

*Figure S7: ^{13}C-NMR relative to compound **2**.*

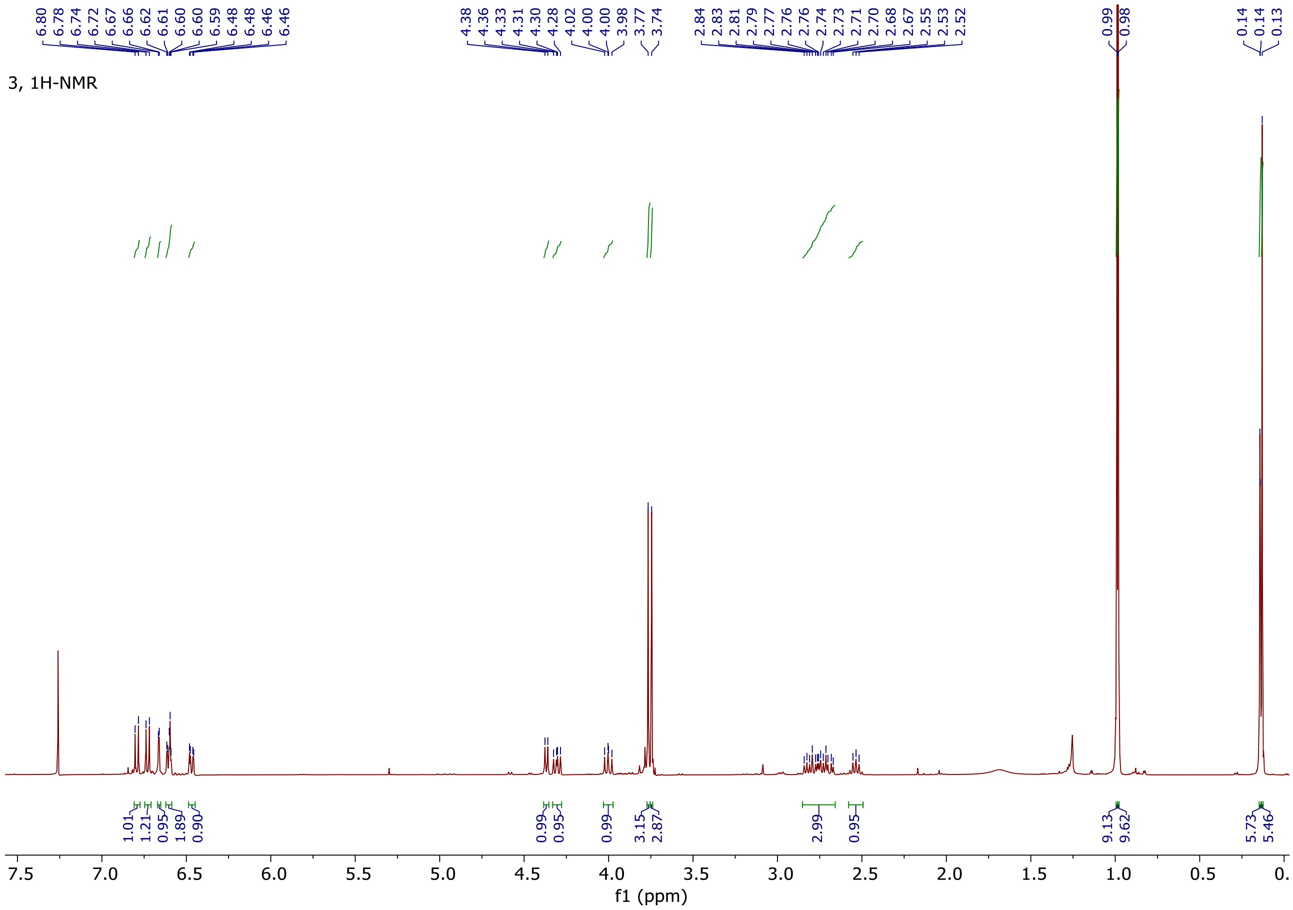

*Figure S8: ¹H-NMR relative to compound **3**.*

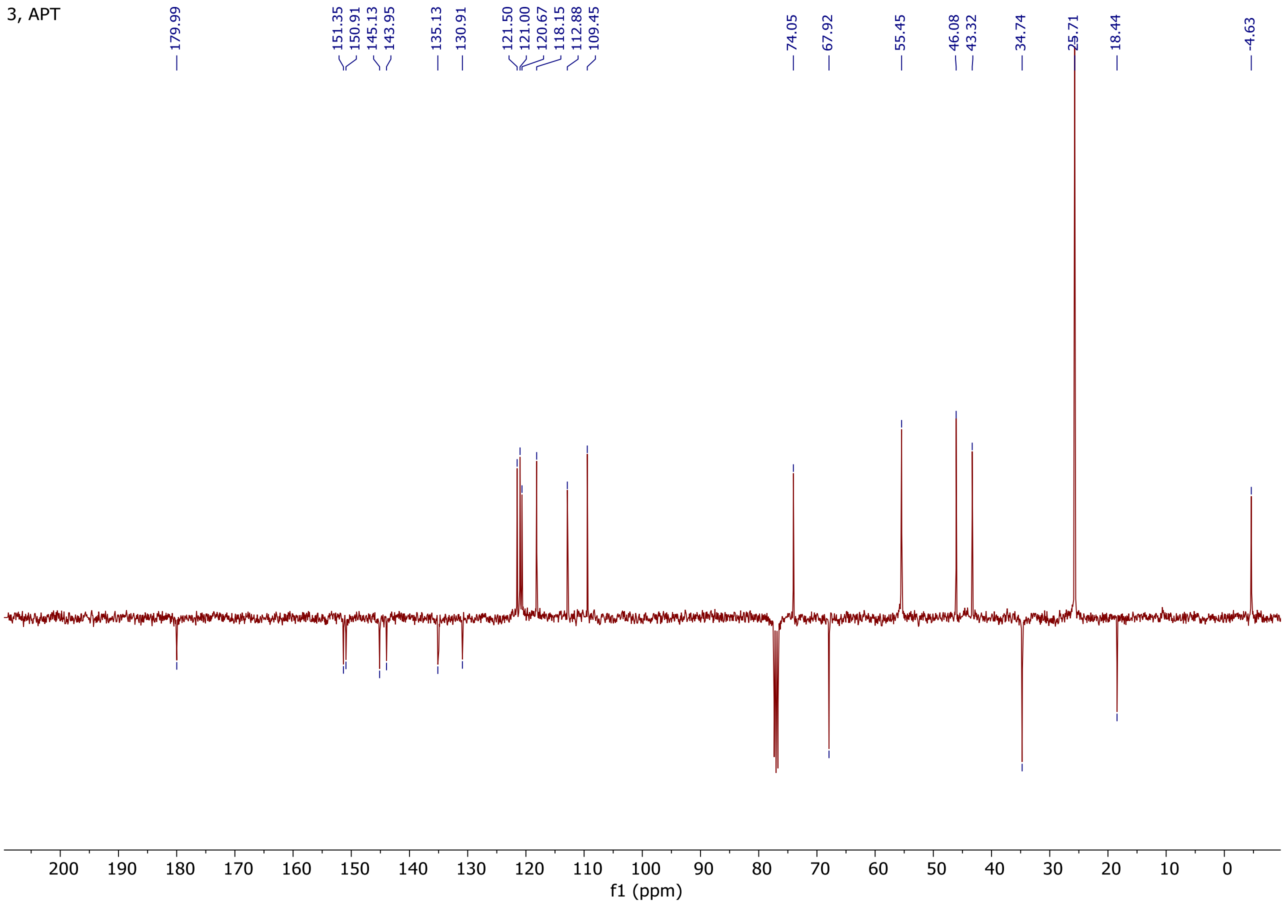

*Figure S9: ^{13}C-NMR relative to compound **3**.*
